# Realfooders Influencers on Instagram: From Followers to Consumers

**DOI:** 10.3390/ijerph18041624

**Published:** 2021-02-08

**Authors:** Javier Gil-Quintana, Sonia Santoveña-Casal, Efrén Romero Riaño

**Affiliations:** 1 Department of Education, National University of Distance Education, 28040 Madrid, Spain; ssantovena@edu.uned.es; 2 Faculty of Engineering, Autónoma University of Bucaramanga, Bucaramanga 680003, Colombia; eromero21@unab.edu.co

**Keywords:** digital media, health literacy, influencers and native advertising

## Abstract

(1) Background: Realfooders have positioned themselves in social networks such as Instagram by posting photographs of recipes, advises, habits and nutritional behaviours which are advertised as reliable nutritional patterns and by their self-promotion as highly trained people in the field of nutrition which sometimes jeopardises the health of digital citizenry. (2) Methods: In this article, we develop a quantitative study for analysing the influence of selected Realfooders on 2,866,980 followers on Instagram, taking into account channel variables (gender, location, interests and motivations), followers’ variables (engagement, interaction and consumption) and some variables related to the message of 54 posts about breakfast. (3) Results: Selected Realfooders concentrate their followers in Spain, mostly women between 18 and 24 and between 35 and 44 years old who link their interests on food to the cult of the body and recreational areas. On the other hand, the content generated by Realfooders has been increasing its impact using advertising and marketing techniques for awaking consumer’s interest. (4) Conclusions: Educational and social agents are facing the challenge of low health literacy in young population. Therefore, it is necessary to design and implement strategies for developing critical thinking that allow them to assess the content generated by Realfooders and identify which recommendations can be harmful or beneficial to their health.

## 1. Introduction

Social networks are considered spaces of social influence, by which actions, thoughts and even feelings are influenced by others connected in the network [1]. Among different types of influence, there is one called normative social influence, which is defined as the “need to please others” [2]. The normative influence directly depends on the degree of relevance of individuals: the greater the relevance, the greater the influence on the group. This degree of relevance is caused by stronger links between nodes and proximity [2].

Hence, it is known that everything that is said or done in the network tends to spread, influencing others’ behaviour depending on the degree of connection between those involved. The greatest degree of influence is found on the first degree of separation (“friends”), which decreases in the second degree of separation (“friend’s friend”) and declines even more in the third-degree (“friend’s friend’s friend”) [3]. The influence can be observed in very different areas such the tobacco consumption, voting intention and, among others, habits related to food.

Numerous studies relate social networks to health status and the influence within the group and among peers. They have been developed from a structural and/or relational approach, covering a wide field of study [4]. It has been stated that having diverse networks reduces the risk of suffering heart problems [5] and facilitates better physical condition [6] and mental health [7]. However, it has also been found that networked influence leads to health-damaging behaviours. There is evidence of an obesity epidemic, referring to the capacity for spreading obesity through networks [3]. It was found that if your friends (first degree of separation) are obese, you have a 45% probability of being obese and this percentage decreases in further degrees of separation to zero at the fourth degree [3]. Among other studies, it has been observed that network contacts have an influence in alcohol consumption [8], HIV-related risk behaviours [9] and smoking [10,11]. Moreover, it was observed that the influence is greater if the contacts who smoke and with whom we have a relationship are more popular [12]. Additionally, popularity in social networks has been related to insufficient levels and shorter duration of sleep in girls, whereas isolation in social networks has been related to insomnia symptoms in boys [13].

In social networks such as Instagram, all kinds of topics can be found related to these healthy habits, movements and posts about anything, including food related topics, such as diets without protein and hyper protein diets, radical vegans, rawism or diets on food supplements with high or low chemical composition. In these digital spaces, links are established between a group of individuals under the midst of social communication networks, being adolescents and young people the most vulnerable to its influence. However, another movement which advocates for healthy eating is increasingly more present in social media. Some influencers, who nowadays are leaders for young generations, show themselves as Realfooders influencers focusing on the “healthy” field of nutrition and influencing the dietary patterns of its young followers [14]. It was from the fight against ultra-processed food that “Realfooding” was born. This movement, led since 2017 by Carlos Ríos, has exposed the interests of the food industry, gaining followers and making enemies at the same speed. Realfooders have invaded social networks, such as Instagram, with photographs of recipes, advice, habits and “healthy” eating routines and “reliable” nutritional guidelines, and they position and advertise themselves as agents with great training in the world of food and nutrition. “Realfooding” was devised as a necessary movement with noble principles, but it may have been taken to an extreme that is not coherent with this, and therefore, their recommendations are not always completely healthy. For this reason, there are concerns about healthy eating in this context, such as orthorexia, a disorder defined by Steven Bratman [15] which consists of an obsession with healthy food, a pathology that can lead to isolation. It has been observed that some food-related behaviours, if taken to the extreme, can be tremendously harmful to health.

The content generated by these influencers in the social networks of the post-digital era [16] can foster an informed, committed and critical public, capable of engaging in the processes of dialogue for improving understanding in the field of nutrition. However, depending on the published content, it can also produce anomalies, encourage prejudice, generate a loss of meaning in life, produce anomie and cause damage to people, especially the most vulnerable [17]. This jeopardizes the public health system itself and the adequate practical implementation of basic health principles: autonomy, charity, justice and non-maleficence [18]. Moreover, the content posted by Realfooders influencers in social networks can be used to exacerbate the benefits of some products or services, create confusion on the scientific information available, transform the consumer into a submissive one [19], cause an increase in inequalities and underestimate or ignore the risks and costs derived from a product or service [19].

It has also been observed that in order to convince, but also to persuade or deceive, influencers rely upon a language of persuasion [20], traditional mechanisms and means of communication and the use of celebrities, research institutes and/or health sector professionals to generate confidence in the products they sell and the services they offer [21]. The confluence of the roles of content producer and consumer (the so-called prosumer) also poses ethical dilemmas since it can confuse people when interpreting or assessing the reliability of information [22]. Since influencers are massively influential and its primary role in social networks is to build the identity of adolescents and young people [23,24,25], these facts are worrying for public health.

The behaviour and tendency to interact on influencer accounts depends on multitude of variables, from individual differences [26] to aspects such as the credibility of the source. In this study, we start from the definition of celebrity credibility as the degree to which it is seen as a source for acquiring experience and knowledge regarding a given product or service [27]. Credibility has been studied from multiple perspectives, from the quality of the argument and its persuasiveness [28] to the trustworthiness, attractiveness and expertise of the communicator [27]. Credibility influences the intention to share a content and interact with the celebrity, but if followers perceive that the influencer’s behaviour is mediated by advertising, this can have a negative impact on their attitude and intention to share that content [29] and, it is probably because the influencer’s credibility is diminished [30]. The decrease in the influencer’s credibility may decrease the intention to interact, for example, if it is perceived that the influencer is making money from advertising [31], because of a lower quality of content, originality, creativity and uniqueness, characteristics highlighted as fundamental for thought leaders on Instagram [32].

Our study is projected in this line of thought, seeking to analyse the processes of coalition, groupings, channels of influence, receptors and messages related to the field of nutrition of Spanish Realfooders in the Network Society [33], with its 2,866,980 followers (data collected in May 2020). Our study is based on the following objectives:

- Objective 1: Understand the impact of the main Spanish Realfooders through the social network Instagram on Spanish youth, according to their location, age, gender, interests and motivations.
- Objective 2: Analyse the strategies, interests, positioning and empowerment of digital marketing used by Spanish Realfooders to position themselves as opinion leaders in the field of nutrition and healthy lifestyle.

## 2. Methodology

Our research is a quantitative study from which descriptive results are extracted: analysing a composite of posts, interactions, activity and exchange in digital scenarios. The internet is conceived in this study as a space of interaction accessible to ethnographic research since what happens on the internet are social interactions [34]. Regarding the quantitative ones, which aim to contrast theories aligned with the positivist paradigm, we focus on the study of observable facts and behaviours, reducing them to simple units to operationalise and measure them from objectivity and neutrality. The quantitative studies played a key role in this research by providing a systematic analysis to compare different Instagram profiles, interpretative studies enabled deeper research about their repercussion on the daily lives of the 2,866,980 Instagram followers of these Realfooders. Analysing the interactions of these heterogeneous groups of followers allow us to identify harmful content for health.

### 2.1. Sample

This study is a descriptive one which takes as reference nine influencers that identified themselves as Realfooders on the social network Instagram. We established the following stages as criteria:

1. Conceptualize the type of relationships we need;
2. Determine whether we need networks based on a single or several links;
3. Building the list of names;
4. Map the links between the influencers and their followers;
5. Commitment, loyalty and impact on the followers.

The non-probabilistic sample was therefore selected by accessing the ranking of Spanish Realfooders in which they were positioned as influencers: Paloma Quintana (48.83 k), Marco Ortiz (162.40 k), Inmifit (25.66 k), Futurlife (359.94 k), Fit_Happy_Sisters (462.48 k), Carlos Ríos (1.44 M), Blanca Nutri (259.12 k), Ana Cruzado (5.21 k) and Alex Yanez (103.34 k). The reason for choosing Realfooders from Spain is because of it is the nationality of the institution and of the research group promoting the study. From this space for research, participation and dialogue, the influence of these influencers on Spanish youth was elucidated through the experiences lived in their environment. The influencers chosen as sample are between 30 and 40 years old and their profiles present personal photos that show a well-groomed physical appearance and a worked gym body. The participants included in the study were the 2,866,980 followers of Realfooders and this sample was beneficial due to its homogeneity in the content that these influencers generate.

Our main interest was not only finding the profile of the members of these networks of influencers and their impact, but also knowing the properties of their personal networks, such as its internal structure, their motivations and the interests of the companies behind certain posts. After describing the impact of the selected influencers, we developed a visual analysis of the content of the 54 posts that the different producers share related to the breakfast. The reason for this selection is because of the consideration of breakfast as a fundamental part of the daily diet and, for this reason, among the publications made by the influencers we have selected following this criterion. We analysed 9281 words and 57 images or audio-visual productions.

Since this is a study of social networks, we are aware that some data can be lost when analysing the common content. However, this selection allows us to build specific knowledge about the content to which followers are exposed by Realfooders.

### 2.2. Procedures

#### 2.2.1. Channel Variables

Realfooder’s personal network has been structured as the first order zone. From this channel, it was possible to obtain information as it is the immediate social environment of influence of the chosen profile. From this personal network, the immediate channels were studied, paying special attention to the number of followers, assessing the popularity of the profile and considering variables such as age, gender, location, interests and motivations.

#### 2.2.2. Followers’ Variables

The main characteristic of the personal network is that people interact through a series of links that come from the activities in which they are involved in one way or another. Actors from a Realfooder’s network are linked by multiple relations through the interest in the field of nutrition. Moreover, this social network produces circuits of influence and commitment between the influencer and his/her followers, which shapes their personal situation by influencing their behaviour, opinions, well-being, health and consumption patterns.

#### 2.2.3. Message Variables

Finally, the influence between Realfooder and their followers is caused by their engagement, thanks to the content produced uninterruptedly in their profile. Videos, stories, slogans, images and other audio–visual productions shape the message produced by the influencers, using media strategies and digital marketing to influence consumers’ dietary patterns. The messages we analysed determine people’s attitudes towards popular nutrition, which can build healthy or unhealthy behaviours in social networks.

### 2.3. Statistical Analysis

The analyses presented within the results include network measures and descriptive statistical measures. We used normalized engagement data to develop measures that indicate the influencer’s ability to generate effective interactions and foster creativity with their followers. To obtain them, the frequency values extracted from the “Influencity” platform were divided by the number of followers. This allows us to establish a measure of interaction effectiveness beyond the nominal quantities observed.

In order to describe the relevance of the topics within the network of influencers’ interests, the degree centrality is estimated for each topic. The degree of centrality is the most used measure of influence within social network analysis. In a network, degree centrality refers to a node’s measure, which determines its relative importance and it is defined as the number of direct connections of a node (node’s neighbourhood). In addition, network maps were developed for identifying the links between topics, countries and influencers.

### 2.4. Instruments

Two software packages were used to process quantitative data and obtain the results. First, we used the “Influencity” software, through which we measured the number of followers (followers of the influencer); the engagement (degree of commitment established with the followers); earned media (income obtained from advertising in dollars); average interactions (interaction rate); followers quality (in %) and the distinction between the number of Doubtful and Nice followers, average interactions data (videos views, likes and comments), influencers’ interests from theme ranking, hashtags and its percentage, mentions and its percentage; influencer brand affinity, audience age and gender percentage, top audience countries and cities, audience interests and audience brand affinity. This platform has given us access to influencer marketing data that we have subsequently analysed based on our research. The “Influencity” software has different search criteria with which to filter influencers and discard fake profiles; it also makes it possible to manage companies’ campaigns that use influencer marketing in their marketing policies. Second, the interpretation of this data was conducted using the software UCINET (Analytic Technologies, Harvard, MA, USA).

## 3. Results

### 3.1. Category 1: Channel Variables

In the first category, we show the number of followers, assessing profiles’ popularities, taking into account variables such as age, gender, location, interests and motivations. We analyse what information each user shares, what recommendations they make and what influence their opinion has on their social network. This analysis may contribute to improving marketing strategies to achieve their objectives and empowering the users of the health system to increase their autonomy and capabilities; optimizing processes, increasing the quality of care; improving forecasts and results related to the prevention and eradication of diseases, etc.

#### 3.1.1. Gender and Location

**Figure 1:**
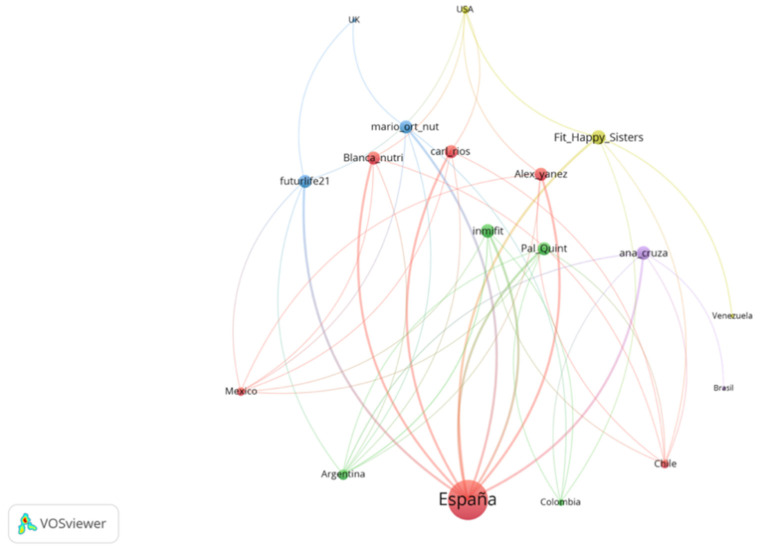
Audience concentration network by country.

**Figure 2:**
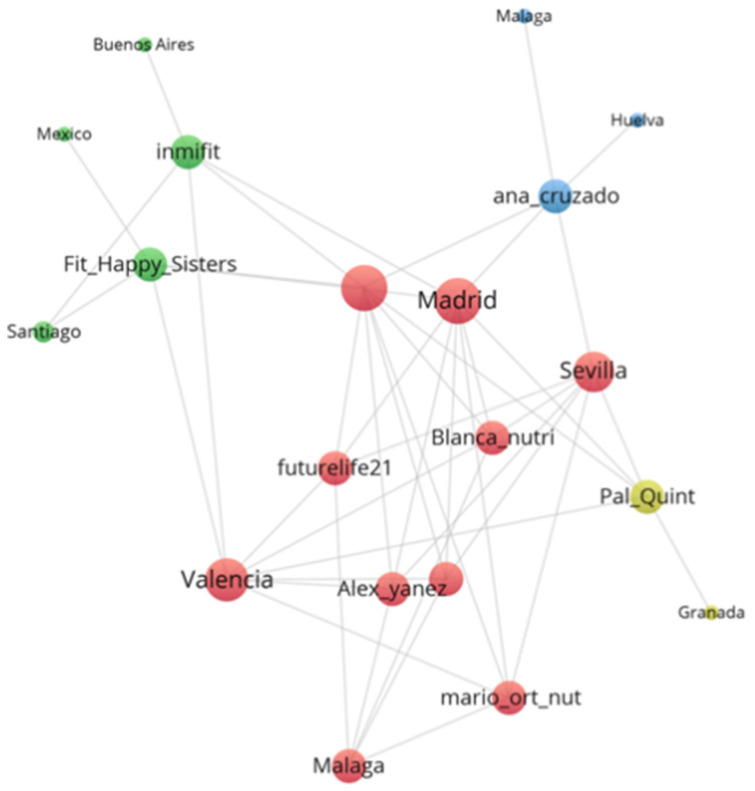
Audience concentration network by city.

The relationship between the selected influencers and the location (countries) where their followers are located is shown in Figure 1. In this figure, bubbles’ size is proportional to the number of followers in each country. The arcs or links indicate the existence and intensity of connections between influencers and followers. All the Realfooder focus their impact within Spain and in Spanish speaking countries. This is not surprising due to the origin and language of the contents analysed. However, two influencers showed some followers’ presence in English-speaking countries, specifically in the United States and the United Kingdom. In addition, the average number of cities where the Realfooders analysed exert influence is 18.87. More specifically, we also observed that the top five of cities with most followers are Spanish, as shown in Figure 2. Madrid, Barcelona and Valencia are home to the most followers and other cities, such as Seville, Malaga, Huelva and Granada, are also important in this regard. From this analysis, it was observed that two Realfooders exert influence over cities outside Spain: Inmifit (Buenos Aires and Santiago); Fit_happy_sisters (Santiago and Mexico City).

**Figure 3:**
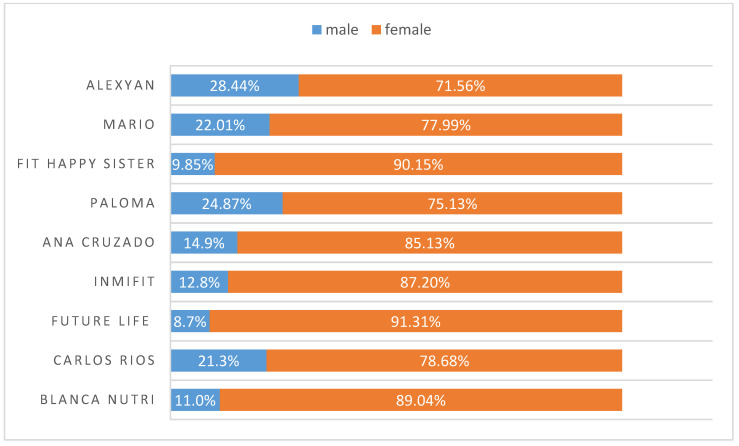
Gender ratio of influencer’s audiences.

The audience gender ratio is shown in Figure 3. The average male audience ratio of the nine influencers analysed is 17.09%. Paloma Quintana (a woman) exhibits the highest percentage of male audience (24.87%), followed by Mario Rincón (22.1%) and Carlos Ríos (21.32%). Hence, the audience of these nine influencers is mostly female, 82.98% on average. In addition, the influencers with the greatest capacity for interacting with diverse audiences are Paloma Quintana and Mario Rincón.

#### 3.1.2. Interests and Motivations

**Figure 4:**
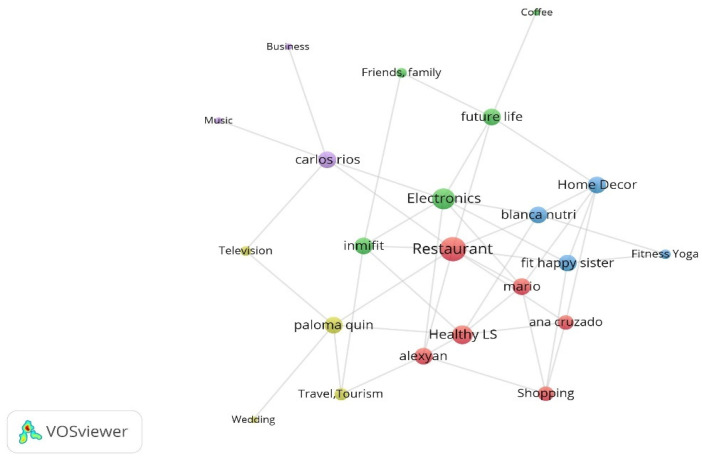
Concentration of the influencers’ interests.

The interests of the nine influencers analysed can be categorised in thirteen topics. In order to identify them, the interests of most Realfooders is summarized in Figure 4. Topics that concentrate influencers’ interest are located in the centre of the network and others of lesser interest are located in the periphery.

The degree centrality (DC) was used to quantify the relevance of the topics within the network and it is calculated as the number of links connected to a node. According to this network measure three topics are identified as being of influencer’s interest: Restaurants, Food and Grocery (DC = 9), Electronics and Computers (DC = 7) and Healthy Lifestyle (DC = 6). The only interest shared by all influencers is Restaurants and Food and Grocery. Electronics and Computers stands out among the most frequent interests; however, this is not related with food topics. On the other hand, audience’s interests are focused on three topics: (i) Restaurants, Food and Grocery (DC = 9), (ii) Travel, Tourism and Aviation (DC = 9) and iii) Friends, Family and Relationships (DC = 9). By comparing the interests of influencers and audiences, matchings are found on topics such as (i) Restaurants, Food and Grocery (9), as expected. However, divergence is identified on (ii) Travel, Tourism and Aviation and Friends, Family and Relationships. As influencer’s interests, only one of the audience topics is related to food. Two topics complement the thirteen most frequent themes within influencers: Camera and Photography and Clothes, Shoes, Handbags and Accessories.

### 3.2. Category 2: Follower’s Variables

#### 3.2.1. Engagement and Interaction

In this category, we analyse audience engagement, which encompasses likes, shares, comments, brand mentions, mentions and profile visits.This variable is mediated by influencer’s characteristics, including credibility since followers assume they are experts on the subject, attractiveness (since it generates emotional or physical connections on followers that can lead to change dietary perceptions) and real motivation, which transmits followers’ honest reasons that are not involved with vested interests and the ability of being empathic with followers, which gave Realfooders greater chances at persuasion.

In this study, we quantify engagement through two standardised interaction indicators, “I like” and “comments”, which are summarized in Table 1. These indicators allow us to estimate an influencer’s ability to achieve effective interactions with its audience or the effectiveness of their posts. The average of the “normalised likes” is 1.95%, while the average of “normalised comments” is 0.22%.

The list of influencers, ordered in descending order by the number of followers on Instagram, is shown in Table 1. In this table, columns exhibit the number of likes and comments followed by its normalised values. The influencer with the highest normalised value of likes and comments is Ana Cruzado. On the other hand, Carlos Rios exhibits the highest values for the number of followers and likes, while Blanca Nutri shows the highest number of comments. These values indicate the influencers’ credibility, since they show the level of interaction they achieve from their posts. According to the values estimated in Table 1, this level of credibility is inversely proportional to the number of followers. The greater the number of followers, the closer the interaction values are to the average of the set measured through “likes.” From the values of “normalised comments”, it is evident that there is an inverse relation between the number of followers and the comments.

#### 3.2.2. From Followers to Consumers

At this point, we analyse Realfooders’ sales, which turns followers into potential customers. Followers cannot always recognize the authorship of contents to which they are exposed and differentiate if those posts are designed with healthy criteria or with advertising purposes. However, the algorithms used in this study take into account the advertising contracts established with companies and the individual and real-time data of user’s personal behaviour (information searches, contact networks, history of commercial transactions, etc.).

**Figure 5:**
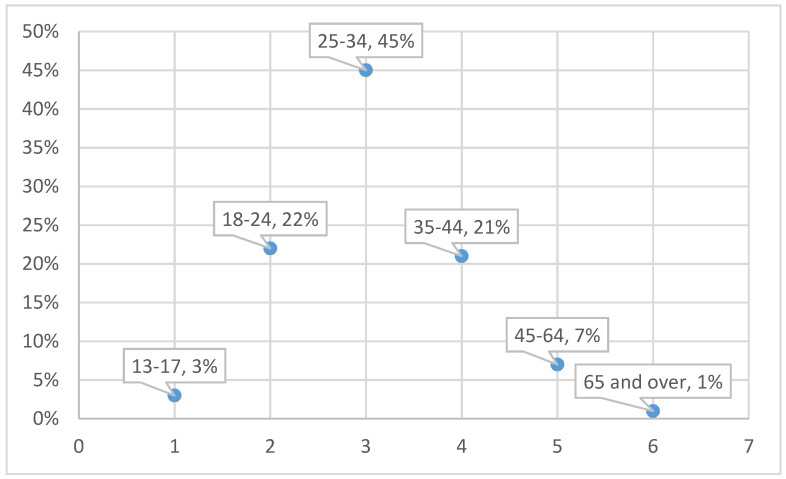
Age groups of Realfooders.

**Figure 6:**
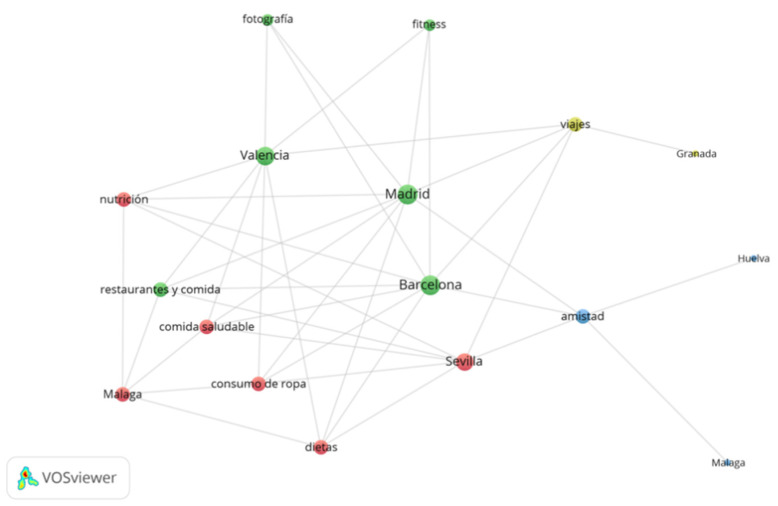
Network of relations between topics and cities.

As shown in Figure 5 and Figure 6, we found that the main targets of these influencers are young women between 25 and 34 years old, followed by those between 18 and 24 and between 35 and 44. These followers are located in the Spanish cities with more inhabitants and that have similar interests related to the human body, such as clothing consumption, healthy food, diets, nutrition and fitness, which can be complemented with other interests, such as friendships, trips and photography.

### 3.3. Category 3: Message Variables

After making a description of the impact of the selected influencers, we developed a content analysis of the posts made over a week by them:

- We chose the contents of the impact analysis as the most efficient (resources) and effective (scope) based on the breakfast;
- We identified the contents that work better in an organic way and respond from a communicative perspective, taking into account:
  ∘ Attractiveness, generating more or less attraction in the follower.
  ∘ The persuasion model used.
  ∘ Sequence of presentation of small discrepancies advancing progressively.
  ∘ Motivational support, as a persuasive factor for the message.
- We distinguished between those contents published as text (9281 words) or as images and audio-visual format (57), classifying them according to the subject matter.
- We compared those contents published by influencers according to the interests of the followers.
- We analysed the suitability of the dietary patterns generated according to the basic health principles: autonomy, charity, justice and non-maleficence (18).

From the 54 Realfooder’s posts analysed, we observed that the aesthetic component is relevant. The dishes showed in images and audio–visual productions are well placed, carefully prepared and with an appropriate photographic quality as shown in Table 2. In the audio–visual material, we also observed that there is an interactive dialogue between followers and Instagramers, using advertising strategies. It is important to highlight that Mario Ortiz’s posts are “stories” with recipes from other influencers; he does not generate recipes but only the image, which is why we do not have several interactions from his followers either.

From the overall assessment we made of content generated by the selected Realfooders, we observed that the first approach of their profiles is clearly to share recipes, food and the creation of dishes considered by them as “healthy.” In the case of the influencers with most followers, we observed that the posting trend changes at the same time as its impact increased, moving away from long texts in their posts to publishing in their profiles advertising posts with great motivational and persuasive content and selling products related to different brands using marketing with slogans, audio–visual productions, images and other types of illustrations that awaken the interest of consumers. From this perspective, other research lines are opened from the field of health for assessing if Realfooders offer menus that are typical of marketing influence and if they provide a suitable nutrition to those that follow these “dietary patterns”.

## 4. Discussion

Educational, health and social agents must be aware of the dependence that young citizens have on the capitalism of social platforms [35] and how their identity is constantly being sold in the form of algorithms [36]. Influencers in the post-digital era use their image to attract followers, engage and make them consume the products and services of its promoting brands. This whole network of influencing marketing [37,38], which is developed from social networks such as Instagram, is the key to ensuring the success [39] and the acquisition of economic benefits [40] of these opinion leaders and creators of the social imaginary [41]. This study on Realfooders support previous findings pointing out that social networks and their connections expose people to dishonest, aggressive, altruistic and collaborative attitudes at the same time, all of them supported and transmitted through connections [42]. On the other hand, studies that highlights the influence of networks on behaviours directly related to health, such as the tendency to obesity [3], found that the most popular network actors are more influential in the tendency to smoke [12].

In our research, we were able to verify how the selected Realfooders concentrate their followers in Spain and Spanish-speaking countries and only two of them are positioned in the United States. In Spain, the cities with the largest number of followers are Madrid, Barcelona and Valencia, followed by Seville, Malaga, Huelva and Granada. Other cities stand out at the international level, such as Buenos Aires, Santiago and Mexico City, in which are Blanca Nutri in the former two and Fit_happy_sisters in the latter ones. The divergence in the gender ratio of the audiences can be partially explained by the influencer’s interests since they are less related with the interests and motivations of the male audience. Hence, these influencers are strongly positioned among women between 18–24 and 35–44 years old. These followers have common interests related to body worship (clothes, healthy food, diets, nutrition, fitness, etc.) and other recreational interests (friendship, photography, travel, etc.).

The credibility of influencers has been measured by the degree of interaction they achieve from their publications. An interesting point is that followers tend to interact less (make fewer comments), the more followers the influencer has. It is not possible to reach a conclusion about the causes of this fact, since there are numerous variables that may be influencing this behaviour, from the perception, by followers, that the influencer is obtaining economic benefits and, therefore, their credibility decreases [31], individual differences [26] or, among other reasons, quality of content, originality, creativity and uniqueness, characteristics highlighted as fundamental to be an opinion leader on Instagram [32].

The level of credibility of the Realfooders increases as the number of interactions and followers increases. Our study observed a trend in published content, the impact of which increases when highly motivational and persuasive content is included, using advertising marketing that generates excitement and interest on the part of the consumers.

Nutrition is a multidisciplinary science and therefore, it is a field shared between doctors, nurses, veterinarians, biochemists, biologists, etc. Hence, it is relevant to highlight that the purposes and interests of each group are different, so it is necessary to differentiate between scientific evidence and marketing, or between patient and consumer, for example.

Among the posts analysed related to the breakfasts, we observed some dangerous statements or recommendations made by Realfooders, for example, in the case of the intermittent fasting. Studies confirm that 5–8% of one’s weight can be lost between 3–6 months; however, weight loss can be much less than these values. Intermittent fasting is very inflexible, which can prevent one from developing routines and generates a risk of self-sabotage [43,44]. Besides, a false and dangerous message is spread. It is stated that after fasting for a few days, people can eat any food, as in the case of a weight-loss diet. These claims could be classified as “fattening training”, since Realfooders disregard here that what is required to lose weight is following general health recommendations and developing healthy habits with suitable eating patterns according to the body characteristics of each person. In conclusion, there is no scientific evidence to support that permanent weight loss can be achieved through intermittent fasting.

On the other hand, from the analysis of Realfooders’ posts, it was observed that they are based on fad diets and advertise trendy food or brand-products as the unique form for the assimilation of nutrients which are already present in any healthy diet. Moreover, some posts include narcissistic self-promotion, criticism without scientific evidence and promoting products without a clear nutritional basis.

Realfooders’ objective of promoting healthy eating habits exhibits several challenges. The first one is the current share (24%) of young people (between 13 and 24 years old) in the Influencer’s audience. According to the World Health Organization (WHO), 39% of adults over 18 years old (39% men and 40% women) were overweight. The audiences’ gender ratio of Spanish Realfooders shows that on average less than 18% of the audience is male. These figures suggest an opportunity to design strategies to increase the penetration on this audience.

Educational, health and social agents must be committed to social transformation through teaching health education for developing a citizenry that “is endowed with action, therefore, it is an active agent, builder and transformer of the environment in which it develops and lives” [45] pp. 46. This type of social dynamic implies a reflexive awareness [46,47] for mobilization [48], in which people become aware of social problems and develop collective solidarity through health literacy for avoiding the manipulation and inadequate use of information.

For these reasons, it is necessary to improve the regulation of information published on social networks for helping citizens to distinguish which come from food experts and which come from untrained people in the field of health and nutrition. In addition, it is required to promote formal and informal health education for training consumers to make decisions based on verified knowledge.

## 5. Conclusions

Social networks are social spaces where multiple topics intertwine. In fact, it has been observed that Instagram expose users to information related with healthy habits from different perspectives based on unverified and unfounded information, such as free protein and hyper protein diets or other influencers that promote food supplements at all costs.

Realfooders establish strong or weak links with other Instagram users. In their networks, they spread not only information, but also thoughts, ideas and emotions, which exert social influence on several degrees depending on their popularity with a stronger effect on the behaviour of the younger population.

Realfooders have taken advantage of this digital opportunity and have used Instagram to become influencers on the life of young citizens. When these influencers post false or unverified information, they can lead people to develop unhealthy behaviours. Obesity, eating disorders or unhealthy eating habits are the new challenges that health education must face.

However, the influence on the behaviour of users of social networks is a very complex area that needs to be addressed from different approaches. The socialization process involves a set of social variables (such as cohesion and affiliation processes), cognitive variables (collective intelligence, critical and reflective thinking) and emotional influence processes [42]. Critical thinking is a basic ability to discriminate false information from that which is not, to detect and manage social problems efficiently through a reflective process and adequate socialization and communication that facilitate the construction of knowledge [49]. And it is here where education plays a major role. In this article, health literacy and the development of critical thinking are the proposed strategies to face this challenge. A critical attitude towards influencers is a way to avoid the development of risky behaviours related to food. This educational process will allow the followers of Realfooders, as well as citizens in general, to develop processes of interaction and reciprocal communication, generate influences, feelings of affiliation and cohesion and forge healthy hyperconnected networks that together shape a safer digital society.

The study was based on the analysis of Spanish accounts, so in order to generalize the results obtained, it is convenient to replicate it with a wider sample of international Instagram accounts of Realfooders.

As a result of this research, new questions have arisen to develop subsequent studies such as: What network structure do the Realfooders form with their followers? Is there a relation between strategic positions in the network and the change of perceptions and/or behaviour? Therefore, identification of the structure of generated networks and calculation of the distance between Realfooders and their followers required to influence them effectively are tasks to address in future studies. From this perspective, other research lines are opened for the field of health, for example, assessing if Realfooders offer menus that are typical of marketing influence and if they provide a suitable nutrition to those that follow these “dietary patterns”.

We are able to verify the urgent need to educate adolescents and young people on health topics for developing a critical thinking and attitude towards the content produced by Realfooders. We also highlight the positive role that these influencers can have in health education, as intermediate leaders, if their messages are in line with the parameters established by the World Health Organization (WHO). From the educational, health and social agents, a process of health literacy should be promoted, avoiding the tendency to fall into diseases and the obsession for healthy food that causes the loss of an optimal state of health.

## Figures and Tables

**Table 1:** Normalized indicators of the interaction of the Realfooders.

Realfooder	Followers	Likes	Comments	Normalised Likes	Normalised Comments
Carlos Rios	1,440,000	17,360	411	1.21%	0.03%
Fit happy sister	462,480	6330	160	1.37%	0.03%
Future Life	359,940	7180	251	1.99%	0.07%
Blanca Nutri	259,120	8380	1290	3.23%	0.50%
Mario Ortiz	162,400	2250	104	1.39%	0.06%
Alexyan	103,340	551	47	0.53%	0.05%
Paloma Quin	48,830	649	75	1.33%	0.15%
Inmifit	25,660	302	8	1.18%	0.03%
Ana Cruzado	5210	279	56	5.36%	1.07%

**Table 2:** Posts and interactions of Realfooders.

Posts and Interactions of Realfooders					
Carlosrios					
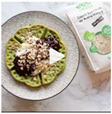	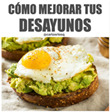	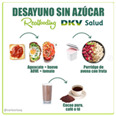	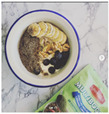	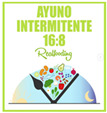	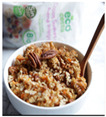
157.232	2531	14.617	11.289	13.490	24.319
Alex Yanez Delacal					
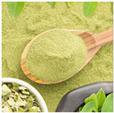	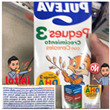	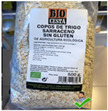	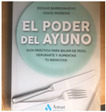	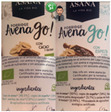	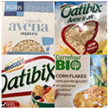
782	1466	824	1854	1050	1948
Ana Cruzado					
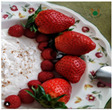	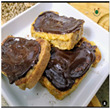	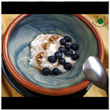	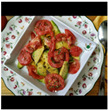	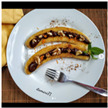	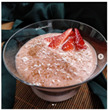
210	246	129	502	288	262
Blancanutri					
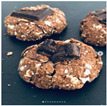	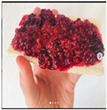	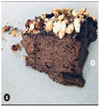	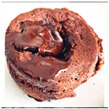	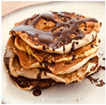	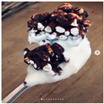
13.511	7216	3684	7057	7503	6414
Fit Happy Sisters					
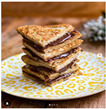	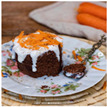	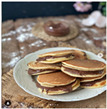	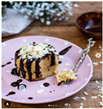	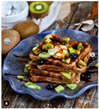	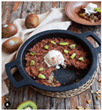
6928	16.603	6824	5528	4967	3528
Futurlife21					
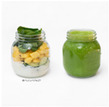	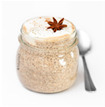	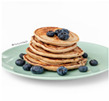	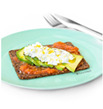	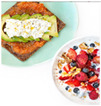	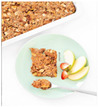
5705	5330	8789	6021	2303	2076
Inmifit					
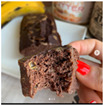	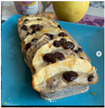	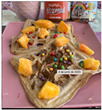	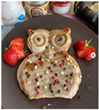	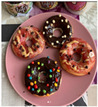	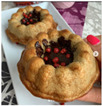
348	335	252	230	238	287
Paloma Quintana					
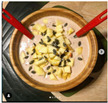	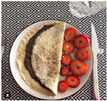	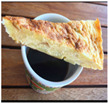	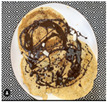	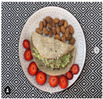	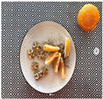
348	630	377	516	644	472
Mario Ortiz					
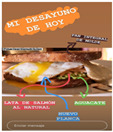	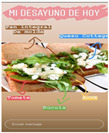	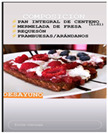	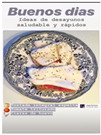	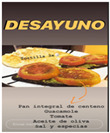	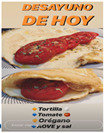

## Data Availability

No new data were created or analyzed in this study. Data sharing is nos applicable to this article.
